# Prevalence and Temporal Trend (2016–2018) of Anaemia among 6–23-Month-Old Infants and Young Children in China

**DOI:** 10.3390/ijerph18042041

**Published:** 2021-02-19

**Authors:** Jing Liu, Junsheng Huo, Zengyan Liu, Jing Sun, Jian Huang

**Affiliations:** 1 National Institute for Nutrition and Health, Chinese Center for Disease Control Prevention, No. 27 Nanwei Road, Xicheng District, Beijing 100050, China; liujingjenny1989@163.com (J.L.); sunjing@ninh.chinacdc.cn (J.S.); huangjian@ninh.chinacdc.cn (J.H.); 2 Fengtai District Center for Disease Control and Prevention, No.0 Kanyang Road, Beijing 100070, China; zengyan_22@163.com

**Keywords:** anaemia, infants and young children, 6- to 23-month-olds, prevalence, temporal trend

## Abstract

Anaemia is a public health problem that can lead to various harmful effects on physical and neurodevelopment in infants and young children (IYC). This study aimed to investigate trends of anaemia and haemoglobin concentration among 6- to 23-month-old Chinese IYC from 2016 to 2018. We used data from the China Nutrition Improvement Project on Children in Poor Areas (CNIPCPA), conducted in 142 national-level poor counties of 20 provinces, autonomous regions, and municipalities from 2016 to 2018. Our study included 103,621 6- to 23-month-old IYC selected by a stratified multistage cluster sampling design. There were 26,303, 33,443, and 43,875 IYC in the survey in 2016, 2017, and 2018, respectively. The overall prevalence of anaemia was 27.0% in the three years. The prevalence of anaemia was 28.0%, 27.2%, and 26.2%, and the mean haemoglobin (Hb) was 11.82, 11.81, and 11.88 g/dL in 2016, 2017, and 2018, respectively. The prevalence of anaemia was highest in 6- to 11-month-olds, and declined with age. There was a gap in the education level between parents. However, the percentage of education improved in the rural areas of China. The prevalence of anaemia decreased significantly in the three years, which shows that prevention and control actions achieved the initial results.

## 1. Introduction

Anaemia, defined as a low blood haemoglobin concentration, is one of the most critical public health problems [1,2]. Although anaemia may result from various causes [3], iron-deficiency anaemia (IDA) is the dominant type, according to available evidence; approximately 50% of individuals with anaemia have iron deficiency [4,5]. Anaemia adversely affects cognitive and motor development, and leads to fatigue and low productivity [6,7]. For infants and young children (IYC), anaemia has irreversible adverse effects on growth and development [8]. It is associated with impaired psychomotor development, impaired cognitive function, and decreased physical activity [9,10,11,12].

Anaemia is a severe global public health problem affecting approximately one-third of the world’s population [8,13]. Children under five years old are vulnerable to anaemia. In 2011, the prevalence of anaemia in children under five years old was 43%, accounting for approximately 273 million children around the world [1]. In China, the prevalence of anaemia among children aged five years or younger was 11.6% across the country, 10.6% in urban areas, and 12.4% in rural areas, based on the China National Nutrition and Health Survey (CNNHS) in 2013 [14]. The first 1000 days of life, from conception to 23 months, is a critical period for establishing a physical, cognitive, and socioemotional foundation for later life [12], and 6- to 23-month-old IYC are a vulnerable group [15]. In some rural areas of China, anaemia prevalence in 6- to 23-month-old children has been shown to be higher than 50%, such as in the poor rural areas of Jiangxi (53.9%) and Qinghai (59.1%) [16,17]. The current anaemia status of 6- to 23-month-olds on a national level is unknown, as the existing data are from 2013 [14]. Furthermore, improvements in IYC nutrition in poor rural areas during the past 20 years, including the introduction of iron-fortified formulas, cereals, soy sauce, and nutrient-dense powder, have led to a decreased prevalence of IDA in the first two years of life [18,19]. However, there has been little research on the national-level trends of anaemia among 6- to 23-month-old IYC in the poor rural areas of China.

Our study focused on the anaemia status of 6- to 23-month-old IYC in poor rural areas of China. The objective of this study was to provide an update on the prevalence of anaemia and to examine the trends of anaemia and haemoglobin concentration among 6- to 23-month-old IYC overall and in subgroups.

## 2. Materials and Methods

### 2.1. Sample Design and Study Population

The China Nutrition Improvement Project on Children in Poor Areas (CNIPCPA) is a yearly national cross-sectional survey of IYC in poor rural areas. All data used in this study were obtained from the monitoring database of the CNIPCPA from 2016 to 2018 [20], which covered 142 counties in 20 provinces, autonomous regions, and municipalities. Eligible participants were selected by a stratified multistage cluster sampling design, which included stratification at the provincial level, multistage sampling, probability proportional to size (PPS) sampling, and systematic random sampling. We selected 107,232 6- to 23-month-old participants from 20 provinces in China. Participants for whom data were missing in terms of their identification number, gender, haemoglobin level, or age were excluded from the study. In the end, our research included 103,621 participants aged 6–23 months.

The Ethical Committee of the National Institute for Nutrition and Health of the Chinese Centre for Disease Control and Prevention provided approval to undertake this study (ethical reference number 2014-001). All caregivers of the participants gave their written informed consent.

### 2.2. Data Collection

#### 2.2.1. Questionnaires

We used questionnaires to collect data about the essential characteristics of the IYC, including sex and age. We also gathered information about the caregivers’ level of education, nationality, and occupation.

#### 2.2.2. Hemoglobin Concentration Analysis

We used a portable HemoCue Hb 301 system (HemoCue AB, Angelholm, Sweden) to measure the haemoglobin (Hb) concentration [21]. Blood samples were collected from the fourth finger of the left hand. Fingertip blood was collected with trace chemical reaction tablets (blood tablets). The threshold value of Hb for anaemia diagnosis for IYC aged 6–23 months is 11 g/dL [22]. We determined anaemia by adjusting for the Hb concentration using the altitude adjustment method recommended by the World Health Organization (WHO) [23].

### 2.3. Statistical Analysis

We carried out statistical analysis using IBM SPSS Statistics for Windows, version 26 (IBM Corp., Armonk, NY, USA). Frequencies and percentages are presented for binary or categorical variables, while continuous variables are described by the mean and standard deviation (SD). The characteristics of the parents and IYC were stratified and described by survey year. The prevalence of anaemia, as well as haemoglobin concentration was analysed by age group.

In the analysis, IYC were categorised into the following age groups: 6–11 months, 12–17 months, and 18–23 months. Level of education was categorised into two groups: Graduated from middle school or above (middle/above), and primary school or below (primary/below). The ethnicity of the parents was divided into Han Chinese and other ethnic minorities. Occupation was divided into none (stay at home) and employed. We used the Pearson’s chi-squared (*χ*^2^) test and Fisher’s exact test to compare binary and categorical variables. All statistical tests were two-sided, and a significance level of less than 0.01 was specified.

## 3. Results

### 3.1. Characteristics of the IYC and Parents

A total of 103,621 6- to 23-month-old IYC were included in our study from 2016 to 2018. The characteristics and evolution of the IYC and their parents in 2016, 2017, and 2018 are displayed in Table 1. There were 26,303, 33,443, and 43,875 IYC in the survey for the three years, respectively. The percentage of IYC in the 6- to 11-month, 12- to 17-month, and 18- to 23-month groups was approximately equal across the three years. Over the three years, there were approximately 52% boys and 48% girls. The median maternal and paternal ages were 27 and 29 years in 2016, and 28 and 30 years in 2017 and 2018. The percentage of parents who graduated from middle school or above was approximately 80% and increased year by year. The majority of the population were of Han ethnicity across all years. Nearly half of the mothers were housewives, and more than three-quarters of the fathers were employed.

### 3.2. Trends in the Prevalence of Anaemia and Haemoglobin Levels of IYC.

#### 3.2.1. Trends in Anaemia Prevalence

The prevalence of anaemia was 27.0% among 6- to 23-month-old IYC in the poor rural areas of China overall from 2016 to 2018. The prevalence of anaemia also appeared to decrease with age, with a high prevalence in younger IYC. Table 2 describes the trends and prevalence of anaemia by age groups from 2016 to 2018. The prevalence of anaemia was 28.0%, 27.2%, and 26.2% in 2016, 2017, and 2018, respectively. There were significant differences in the anaemia prevalence rate between different years (*χ*^2^ = 28.484, *p* < 0.001, trend *χ*^2^ = 28.461, *p* < 0.001). The prevalence of anaemia in IYC between 6 and 11 months of age was higher than in the other age groups. Furthermore, the prevalence of anaemia in the 6- to 11-month-old and 12- to 17-month-old groups decreased from 2016 to 2018 (*χ*^2^ = 33.955, *p* < 0.001, trend *χ*^2^ = 33.117, *p* < 0.001; *χ*^2^ = 18.864, *p* < 0.001, trend *χ*^2^ = 18.106, *p* < 0.001). The trend of anaemia in the 18- to 23-month group from 2016 to 2018 had no significant definite direction (*χ*^2^ = 1.755, *p* = 0.416, trend *χ*^2^ = 1.069, *p* = 0.301).

#### 3.2.2. Trends in Hemoglobin

Table 3 shows the trends and means of the Hb concentration among different age groups in 2016, 2017, and 2018. The overall mean Hb was 11.84 g/dL, and the mean was significantly different in each year (F = 39.505, *p* < 0.001, trend F = 39.710, *p* < 0.001). The mean Hb in 2018 was 11.88 g/dL, which was higher than in 2016 and 2017. There was an improvement in the mean Hb of approximately 0.06 g/dL from 2016 to 2018. The lowest mean Hb of the 18- to 23-month group was in 2017, and there was a significantly different, but not obviously linear trend in the three years (F = 5.474, *p* = 0.004, trend F = 0.012, *p* < 0.914). The average Hb levels were higher in older, rather than younger IYC each year. Specifically, the mean Hb was 11.5, 11.78, and 12.13 g/dL in the 6- to 11-month, 12- to 17-month, and 18- to 23-month-old groups, respectively.

#### 3.2.3. Haemoglobin Distribution by Year and Gender

**Figure 1:**
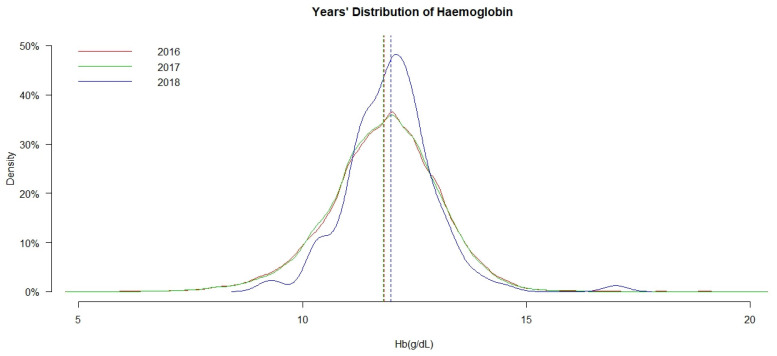
Time trends in the distribution of haemoglobin (Hb) in 2016, 2017, and 2018.

Figure 1 shows the time trends of the distribution of Hb among 6- to 23-month-old IYC in 2016, 2017, and 2018. The coloured lines represent the mean Hb in the three years. There is a clear shift to the right in the density distribution of Hb in 2018 compared to 2016 and 2017. In 2016 and 2017, the mean Hb lines and density distribution curves are similar. In 2018, the density distribution curve is higher than the others, showing more people with higher Hb.

**Figure 2:**
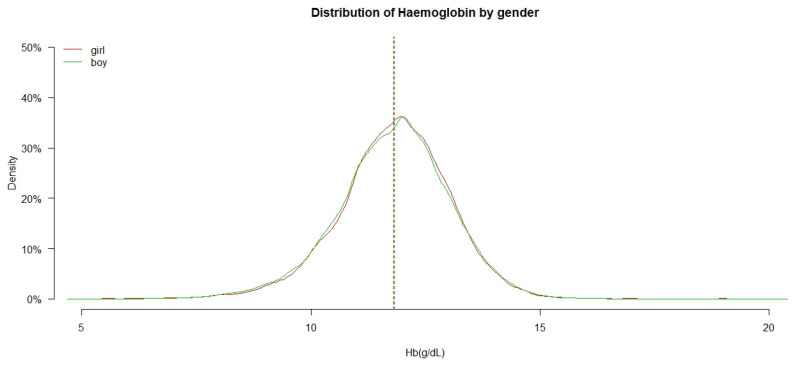
The distribution of haemoglobin (Hb) in boys and girls

Figure 2 shows the density distribution and mean of Hb among 6- to 23-month-old IYC in boys and girls. The color curves show the density distribution of Hb in gender, which were very similar in boys and girls. The colored lines which represent the mean of Hb in different genders almost coincide. The mean Hb of boys and girls was 11.84 ± 1.23 g/dL and 11.85 ± 1.21 g/dL, respectively, and the mean was not significantly different in each gender (F = 5.672, *p* = 0.017).

## 4. Discussion

This study comprehensively updated the national trends in the prevalence of anaemia and the concentration of Hb among 6- to 23-month-old IYC in the rural areas of China. A total of 103,621 6- to 23-month-old IYC were included in our study from 2016 to 2018. The WHO classifies anaemia as a moderate public health problem when its prevalence is between 20% and 40% [2]. Our findings revealed that there has been a significant decrease in the prevalence of anaemia among 6- to 23-month-old IYC in the rural areas of China during the past three years, with rates ranging from 28.0% to 26.2%. It was much better than many developing countries, such as central Asia (64.7%), South Asia (54.8%), and Andean Latin America (62.3%) [13]. The mean Hb in 2018 was 11.88 g/dL. Compared with the means in 2016 and 2017, it increased by 0.06 and 0.07 g/dL, respectively. Overall, the status of anaemia had improved among infants and young children in poor rural areas of China.

The prevalence of anaemia among 6- to 23-month-old IYC in our study was 27.0%, which was significantly lower than the poor rural areas of 10 provinces in 2016 and 2017 (58.1%) [24]. Furthermore, it was roughly consistent with the results of a systematic review of 0- to 3-year-old infants in China from 2010 to 2019 (25.1%) [25]. Our result also showed that the prevalence of anaemia in rural areas reduced. The progress in socioeconomic and educational conditions between 2016 and 2018 observed in the present study may have contributed to this reduction. One of the primary reasons was that the Chinese government formulated many policies targeted to helping people in poor rural areas to lift them out of poverty [26,27]. To improve children’s nutritional status, the Chinese government distributed Yingyangbao (YYB), a nutrient-dense complementary food supplement, to the parents of 6- to 23-month-old IYC in poor rural areas [28,29]. Our research showed that the number of parents who went out to work increased year by year, which may have allowed them to earn a greater salary and to enhance their quality of life. The percentage of parents with a middle/above average education level increased over the years. Previous studies showed that parents with a higher educational level and a greater income had a protective association with anaemia [30,31,32,33].

The prevalence of anaemia in 6- to 11-month-old IYC was found to be the highest among all the age groups, and the prevalence of anaemia decreased with age. Young age is a factor associated with a high prevalence of anaemia, and there is a similar status in many countries [34,35]. Between 6–11 months is also a crucial period to introduce complementary food. However, iron-fortified food and red meat have not been fed in an adequate and timely manner due to religious, economic, and other reasons, which has led to a high prevalence of anaemia [36,37]. In our results, the haemoglobin concentration of boys and girls was not significantly different, and some researchers have found the same results [6,17]. When girls enter puberty, menstruation might lead to lower haemoglobin levels than boys [38,39]. Though the prevalence of anaemia has decreased yearly, it has also been found to be higher in developed countries, such as Canada and the US [8,40,41]. Certain study limitations should also be noted. The numbers of participants were different in 2016, 2017, and 2018. However, this study presents updated data on anaemia prevalence and its temporal trend. The data of our research came directly from the CNIPCPA, which covers all provinces, autonomous regions, and municipalities throughout China that had national-level poverty-stricken counties. Therefore, this study has very good representativeness and is a timely assessment of the trends of anaemia. In the future, intervention measures, such as Yingyangbao for anaemia, need to be evaluated in larger groups of infants. The government should introduce more targeted policies and pay more attention to vulnerable infants to decrease the prevalence of anaemia and improve their growth status.

## 5. Conclusions

In summary, anaemia remains a moderate public health problem in China among 6- to 23-month-old IYC. There has been a decline in the prevalence of anaemia from 2016 to 2018 among this population, showing that the implementation of prevention and control actions from the Chinese government achieved initial results. The government should pay more attention to vulnerable infants and develop policies to relieve the high prevalence of anaemia in rural areas of China where resources are sparse, and they are relatively more impoverished.

## Figures and Tables

**Table 1:** Characteristics of the surveyed children and their caregivers (*n* = 43,374).

Characteristics		Sample (Percentage) (*n* = 103,621)		
		2016 (*n* = 26,303)	2017 (*n* = 33,443)	2018 (*n* = 43,875)
**IYC**				
Month group, *n* (%)				
	6–11 months	7865 (29.9)	10,710 (32.0)	13,763 (31.4)
	12–17 months	9286 (35.3)	11,411 (34.1)	14,731 (33.6)
	18–23 months	9152 (34.8)	11,322 (33.9)	15,381 (35.1)
Sex, *n* (%)				
	Boy	13,701 (52.1)	17,347 (51.9)	22,646 (51.6)
	Girl	12,602 (47.9)	16,096 (48.1)	21,229 (48.4)
**Mother**				
Age (median—Q1, Q3)		27 (24,30)	28 (25,31)	28 (25,32)
Nationality				
	Han ethnicity	17,719 (67.4)	23,688 (70.8)	31,327 (71.4)
	Minority	6281 (23.9)	9652 (28.9)	12,534 (28.6)
	Unknown	2303 (8.8)	103 (0.3)	14 (0.0)
Education				
	Primary/below	5192 (19.7)	5879 (17.6)	7034 (16.0)
	Middle/above	20,907 (79.5)	27,382 (81.9)	36,736 (83.7)
	Unknown	204 (0.8)	182 (0.5)	105 (0.2)
Occupation				
	None	13,409 (51.0)	17,807 (53.2)	22,527 (51.3)
	Employed	12,596 (47.9)	15,387 (46.0)	21,229 (48.4)
	Unknown	298 (1.1)	249 (0.7)	119 (0.3)
**Father**				
Age (median—Q1, Q3)		29 (26,33)	30 (27,33)	30 (27,34)
Nationality				
	Han ethnicity	17,315 (65.8)	23,752 (71.0)	31,366 (71.5)
	Minority	5304 (20.2)	9491 (28.4)	12,416 (28.3)
	Unknown	3684 (14.0)	200 (0.6)	93 (0.2)
Education				
	Primary/below	4139 (15.7)	4727 (14.1)	5808 (13.2)
	Middle/above	21,877 (83.2)	28,426 (85.0)	37,815 (86.2)
	Unknown	287 (1.1)	290 (0.9)	252 (0.6)
Occupation				
	None	5406 (20.6)	6796 (20.3)	7897 (18.0)
	Employed	20,499 (77.9)	26,288 (78.6)	35,713 (81.4)
	Unknown	398 (1.5)	359 (1.1)	265 (0.6)

IYC = infants and young children; Q1 = first quartile; Q3 = third quartile.

**Table 2:** Trends in prevalence of anaemia (%) by age in 2016, 2017, and 2018.

Year	Anaemia Prevalence (%)			
	Overall	6–11 Months	12–17 Months	18–23 Months
2016	7376 (28.0)	2770 (35.2)	2822 (30.4)	1784 (19.5)
2017	9089 (27.2)	3630 (33.9)	3268 (28.6)	2191 (19.4)
2018	11,505 (26.2)	4340 (31.5)	4094 (27.8)	3071 (20.0)
*χ* ^2^	28.484	33.955	18.864	1.755
*p*-value	<0.001	<0.001	<0.001	0.416
Trend *χ*^2^	28.461	33.117	18.106	1.069
*p*-value	<0.001	<0.001	<0.001	0.301
Total	27,970 (27.0)	10,740 (33.2)	10,184 (28.7)	7046 (19.7)

**Table 3:** Trends in haemoglobin concentration (mean ± SD) by age in 2016, 2017, and 2018.

Survey Year	Mean Haemoglobin (g/dl)			
	Overall	6–11 Months	12–17 Months	18–23 Months
2016	11.82 ± 1.25	11.53 ± 1.24	11.75 ± 1.25	12.14 ± 1.18
2017	11.81 ± 1.22	11.55 ± 1.18	11.76 ± 1.22	12.10 ± 1.18
2018	11.88 ± 1.21	11.65 ± 1.19	11.82 ± 1.21	12.15 ± 1.17
F-value	39.505	33.279	12.655	5.474
*p*-value	<0.001	<0.001	<0.001	0.004
Trend F-value	39.710	53.011	17.738	0.012
*p*-value	<0.001	<0.001	<0.001	0.914
Total	11.84 ± 1.22	11.59 ± 1.20	11.78 ± 1.23	12.13 ± 1.18

## Data Availability

The data presented in this study are available on request from the corre-sponding author. The data are not publicly available due to privacy issues.

## References

[1] Stevens G.A., Finucane M.M., De-Regil L.M., Paciorek C.J., Flaxman S.R., Branca F., Peña-Rosas J.P., Bhutta Z.A., Ezzati M. (2013). Global, regional, and national trends in haemoglobin concentration and prevalence of total and severe anaemia in children and pregnant and non-pregnant women for 1995–2011: A systematic analysis of population-representative data. Lancet Glob. Health.

[2] WHO (2011). Haemoglobin Concentrations for the Diagnosis of Anaemia and Assessment of Severity.

[3] Wang M. (2016). Iron deficiency and other types of anemia in infants and children. Am. Fam. Physician.

[4] Petry N., Olofin I., Hurrell R.F., Boy E., Wirth J.P., Moursi M., Donahue Angel M., Rohner F. (2016). The proportion of anemia associated with iron deficiency in low, medium, and high human development index countries: A systematic analysis of national surveys. Nutrients.

[5] WHO (2015). The Global Prevalence of Anaemia in 2011.

[6] Balarajan Y., Ramakrishnan U., Ozaltin E., Shankar A.H., Subramanian S.V. (2011). Anaemia in low-income and middle-income countries. Lancet.

[7] Haas J.D., Brownlie T. (2001). Iron deficiency and reduced work capacity: A critical review of the research to determine a causal relationship. J. Nutr..

[8] Lopez A., Cacoub P., Macdougall I.C., Peyrin-Biroulet L. (2016). Iron deficiency anaemia. Lancet.

[9] Tseng P.T., Cheng Y.S., Yen C.F., Chen Y.W., Stubbs B., Whiteley P., Carvalho A.F., Li D.J., Chen T.Y., Yang W.C. (2018). Peripheral iron levels in children with attention-deficit hyperactivity disorder: A systematic review and meta-analysis. Sci. Rep..

[10] Eden A.N. (2005). Iron deficiency and impaired cognition in toddlers: An underestimated and undertreated problem. Paediatr. Drugs.

[11] Hergüner S., Keleşoğlu F.M., Tanıdır C., Cöpür M. (2012). Ferritin and iron levels in children with autistic disorder. Eur. J. Pediatr..

[12] Pivina L., Semenova Y., Doşa M.D., Dauletyarova M., Bjørklund G. (2019). Iron deficiency, cognitive functions, and neurobehavioral disorders in children. J. Mol. Neurosci..

[13] Kassebaum N.J., Jasrasaria R., Naghavi M., Wulf S.K., Johns N., Lozano R., Regan M., Weatherall D., Chou D.P., Eisele T.P. (2014). A systematic analysis of global anemia burden from 1990 to 2010. Blood.

[14] Fang H., Yu D., Guo Q., Ju L. (2018). The prevalence of anaemia among 0–5 years old children in China. Chin. J. Public Health.

[15] Gupta P.M., Perrine C.G., Mei Z., Scanlon K.S. (2016). Iron, anemia, and iron deficiency anemia among young children in the United States. Nutrients.

[16] Ding X., Zhang F., He Q., Mao Z., Li R. (2016). Nutrition effectiveness of 6-18 months old infants in low-income rural areas in Jiangxi province. Mod. Prev. Med..

[17] Huang Y., Wang L., Huo J., Wu Q., Wang W., Chang S., Zhang Y. (2019). Prevalence and causes of anaemia in children aged 6–23 months in rural Qinghai, China: Findings from a cross-sectional study. BMJ Open.

[18] Huo J., Sun J., Miao H., Yu B., Yang T., Liu Z., Lu C., Chen J., Zhang D., Ma Y. (2002). Therapeutic effects of NaFeEDTA-fortified soy sauce in anaemic children in China. Asia Pac. J. Clin. Nutr..

[19] Li Z., Li X., Sudfeld C.R., Liu Y., Tang K., Huang Y., Fawzi W. (2019). The Effect of the Yingyangbao complementary food supplement on the nutritional status of infants and children: A systematic review and meta-analysis. Nutrients.

[20] National Institute for Nutrition and Health Chinese Center for Disease Control Prevention (2020). The China Nutrition Improvement Project on Children in Poor Areas. Beijing, China. https://ffoiycn.chinanutri.cn/back/Login.aspx..

[21] Ziemann M., Lizardo B., Geusendam G., Schlenke P. (2011). Reliability of capillary hemoglobin screening under routine conditions. Transfusion.

[22] International Nutritional Anemia Consultative Group (INACG), United Nations Childrens Fund (UNICEF) (1998). Guidelines for the Use of Iron Supplements to Prevent and Treat Iron Deficiency Anemia.

[23] WHO (2001). Iron Deficiency Anaemia Assessment, Prevention and Controls.

[24] Chen C., Anish K., Huang Y., Wang Y., Gao Y., Zhou H. (2020). Anemia prevalence and its relationship with dietary diversity in children aged 6 to 23 months in rural China. Chin. J. Reprod. Health.

[25] Zhou J., Liu D., Liu R. (2020). Meta analysis-the prevalence of iron deficiency anemia in Chinese infants aged 0–3 years from 2010 to 2019. Matern. Child Health Care China.

[26] The General Office of the State Council (2014). Suggestions on Further Mobilizing all Sectors of Society to Participate in Poverty Alleviation and Development.

[27] The State Council (2016). The 13th Five-Year Plan for poverty Alleviation.

[28] Huo J. (2017). Ying Yang Bao: Improving complementary feeding for Chinese infants in poor regions. Nestle Nutr. Inst. Workshop Ser..

[29] Wang J., Chang S., Zhao L., Yu W., Zhang J., Man Q., He L., Duan Y., Wang H., Scherpbier R. (2017). Effectiveness of community-based complementary food supplement (Yingyangbao) distribution in children aged 6–23 months in poor areas in China. PLoS ONE.

[30] Zhou X., Fang J., Luo J., Wang H., Du Q. (2017). Factors associated with malnutrition among infants and young children aged 6–23 months in poor rural areas in Hunan Province, China. Chin. J. Prev. Med..

[31] Goswmai S., Das K.K. (2015). Socio-economic and demographic determinants of childhood anemia. J. Pediatr. (Rio J.).

[32] Dey S., Goswami S., Dey T. (2013). Identifying predictors of childhood anaemia in north-east India. J. Health Popul. Nutr..

[33] Xu J., Li Y., Huo J., Sun J., Huang J. (2019). Supplementing fortified soybean powder reduced anemia in infants and young children aged 6–24 months. Nutr. Res..

[34] Vieira R., do Livramento A.R.S., Calheiros M.S.C., Ferreira C.M.X., Dos Santos T.R., Assunção M.L., Ferreira H.D.S. (2018). Prevalence and temporal trend (2005–2015) of anaemia among children in Northeast Brazil. Public Health Nutr..

[35] Gebreweld A., Ali N., Ali R., Fisha T. (2019). Prevalence of anemia and its associated factors among children under five years of age attending at Guguftu health center, South Wollo, Northeast Ethiopia. PLoS ONE.

[36] Aguayo V.M. (2017). Complementary feeding practices for infants and young children in South Asia. A review of evidence for action post-2015. Matern. Child. Nutr..

[37] Duan Y., Yang Z., Lai J., Yu D., Chang S., Pang X., Jiang S., Zhang H., Bi Y., Wang J. (2018). Exclusive breastfeeding rate and complementary feeding indicators in China: A national representative survey in 2013. Nutrients.

[38] Song Y., Wang H.J., Dong B., Wang Z., Ma J., Agardh A. (2017). National trends in hemoglobin concentration and prevalence of anemia among Chinese school-aged children, 1995–2010. J. Pediatr..

[39] Achouri I., Aboussaleh Y., Sbaibi R., Ahami A., El Hioui M. (2015). Prevalence of iron deficiency anaemia among school children in Kenitra, Northwest of Morocco. Pak. J. Biol. Sci..

[40] Looker A.C., Dallman P.R., Carroll M.D., Gunter E.W., Johnson C.L. (1997). Prevalence of iron deficiency in the United States. Jama.

[41] Cogswell M.E., Looker A.C., Pfeiffer C.M., Cook J.D., Lacher D.A., Beard J.L., Lynch S.R., Grummer-Strawn L.M. (2009). Assessment of iron deficiency in US preschool children and nonpregnant females of childbearing age: National Health and Nutrition Examination Survey 2003–2006. Am. J. Clin. Nutr..

